# Effects of the Sequence of Empiric Beta-Lactam and Vancomycin Administration on Clinical Outcomes in Patients with Bloodstream Infection: A Systematic Review

**DOI:** 10.3390/jcm15031024

**Published:** 2026-01-27

**Authors:** Abdulmajeed Alsuwaylihi, Abdulmajeed M. Alshehri, Majed S. Al Yami

**Affiliations:** 1 Department of Pharmacy Practice, College of Pharmacy, King Saud Bin Abdulaziz University for Health Sciences, Riyadh 11481, Saudi Arabia; 2 King Abdullah International Medical Research Center, Riyadh 11481, Saudi Arabia; 3 King Abdulaziz Medical City, Ministry of the National Guard Health Affairs, Riyadh 14611, Saudi Arabia

**Keywords:** β-lactam, vancomycin, administration sequence, bloodstream infection, bacteremia, mortality, clinical outcomes, treatment timing

## Abstract

**Background/Objectives**: Beta-lactam antibiotics (BLAs) and vancomycin have remained the cornerstones of therapy for serious bacterial infections, especially bloodstream infections (BSIs). The clinical impact of administering BLAs before vancomycin on outcomes remains unclear and poorly synthesized. Therefore, this systematic review aims to synthesize the available evidence on the impact of the relative timing of BLA administration to vancomycin initiation on important clinical outcomes in patients with BSIs. **Methods**: A comprehensive search was performed to retrieve clinical studies that evaluated the impact of the sequence of BLA and vancomycin administration on clinical outcomes. Beta-lactam–first group (BLF) included patients who received a BLA before vancomycin, while vancomycin–first group (VF) included patients who received vancomycin prior to BLAs. The systematic review was performed according to the Preferred Reporting Items for Systematic Reviews and Meta-Analyses (PRISMA) statement. **Results**: A total of three retrospective observational studies were included, with a sample size of 29,005 patients, with 24,356 patients in the BLF and 4649 patients in the VF. One study reported that prioritizing BLAs over vancomycin resulted in a 52% reduction in 7-day mortality (adjusted OR, 0.48; 95% CI, 0.33–0.69) and a 55% reduction in 48 h mortality (adjusted OR: 0.45; 95% Cl, 0.24–0.83). Similarly, another study found the BLF strategy was associated with a modest reduction in in-hospital mortality (adjusted OR: 0.89; 95% CI: 0.80–0.99). However, no difference was found in the most recent small, single-institution study that included patients with BSIs. **Conclusions**: The evidence suggests a potential survival benefit for the BLF strategy over VF in patients with suspected or confirmed BSIs. Larger prospective studies are required to confirm the findings.

## 1. Introduction

Beta-lactam antibiotics (BLAs) and vancomycin have remained the cornerstones of therapy for serious bacterial infections, especially bloodstream infections (BSIs) [1,2]. BLAs, which include penicillins and cephalosporins, interfere with bacterial cell wall synthesis and are widely used for both Gram-positive and Gram-negative infections [3]. They are particularly effective against a broad range of Gram-positive and Gram-negative bacteria, with a spectrum varying by agent [1,3]. Vancomycin, a glycopeptide antibiotic, provides its bactericidal activity by inhibiting cell wall synthesis and is only active against Gram-positive pathogens [4]. It is usually reserved for use in methicillin-resistant *Staphylococcus aureus* (MRSA) infections or in patients with severe BLA allergies [4].

Previous studies have explored the impact of adequate empirical antibiotic therapy on clinical outcomes in BSIs. The importance of appropriately early antibiotic administration is well established, and delays in its administration are linked to increased mortality, extended lengths of hospitalization, and higher healthcare costs [5,6]. However, patients with suspected sepsis or bloodstream infection are often started on broad-spectrum empirical therapy, typically an antipseudomonal BLA to cover Gram-negative organisms, and an MRSA-active agent, such as vancomycin, is added empirically in patients with risk factors for MRSA or in settings with high MRSA prevalence pending final culture and susceptibility results. [7,8,9,10,11,12].

Despite the common clinical practice of either initial co-administration or sequential administration of these agents, the clinical impact of administering empiric BLAs before vancomycin on outcomes in patients diagnosed with a BSI remains unclear and poorly synthesized. Guidelines often emphasize rapid, appropriate coverage but provide limited guidance regarding how best to order or optimally time these agents when both are part of the initial strategy [2]. The key clinical question is whether starting a BLA earlier offers any real advantage, such as treating a co-infection, providing better initial coverage before culture results are available, or enhancing vancomycin’s effect, or whether the timing makes no difference or could even be harmful by increasing side effects or delaying the right targeted treatment. Few observational studies have evaluated the impact of BLA administration prior to vancomycin and have shown conflicting findings on the impact of the sequence on mortality [13,14,15].

Therefore, this systematic review is warranted and necessary to synthesize the available evidence on the impact of the sequence of empiric BLA and vancomycin administration on clinical outcomes in patients with BSIs. This will be helpful for providing insights for optimizing clinical practice, guiding empiric antibiotic selection strategies, and possibly enhancing patient care by a balance between the need for early broad coverage and the avoidance of unnecessary drug exposure.

## 2. Materials and Methods

### 2.1. Standard Protocol and Registrations

The systematic review protocol was registered in the International Prospective Register of Systematic Reviews (PROSPERO) with the registration number CRD420251170493 and was performed according to the Preferred Reporting Items for Systematic Reviews and Meta-Analyses (PRISMA) statement (Table S1) [16].

### 2.2. Sources, Searches, and Study Selection

A comprehensive independent literature search was performed in PubMed, Web of Science, and the Cochrane Library to retrieve clinical studies that evaluated the impact of the sequence of empiric beta-lactam and vancomycin administration on clinical outcomes. The comprehensive search was performed from inception until 30 October 2025. Studies included in this review categorized patients according to the sequence of antibiotic administration during the initial treatment of bloodstream infection (BSI). Beta-lactam–first group (BLF) included patients who received a broad-spectrum beta-lactam antibiotic (e.g., cefepime, piperacillin–tazobactam, meropenem, or other beta-lactam agents) before the initiation of vancomycin therapy, while vancomycin–first group (VF) included patients who received vancomycin prior to beta-lactam administration. Studies that did not evaluate the impact of the timing or compare combination therapy to monotherapy were excluded because sequencing is only relevant during the empiric phase. Once pathogens are identified, therapy is de-escalated to targeted antibiotics, and the effect of monotherapy is well-established in the literature for the targeted pathogens. The following search terms were used to screen and extract relevant studies: (“beta-lactam” OR “penicillin” OR “cephalosporin” OR “carbapenem”) AND (“vancomycin”) AND (“bacteremia” OR “bloodstream infection” OR “sepsis”) AND (“timing” OR “empiric therapy” OR “sequential therapy” OR “combination therapy”). The complete search strategy is presented in Table S2. Two investigators (AA, AMA) independently screened and reviewed the relevant studies, and any disagreements were resolved by consulting a third investigator (MSA).

### 2.3. Data Extraction, Risk of Bias Assessment, and Data Synthesis

Two independent investigators systematically collected the relevant details from every included study using a standard data extraction form. The key information that was gathered included the following: first author, publication year, country, study type, specific bacterial organisms, illness/mortality scores, patient age, and the precise definition, type, and incidence of mortality with the reported odds ratios (OR) with 95% confidence intervals (CI). Treatment groups and whether patients were in the BLF or the VF were also collected. The risk of bias for each study was evaluated using the Newcastle-Ottawa Scale (Table S3) by two independent investigators, and any disagreement was resolved by the third investigator [17]. The authors did not perform any additional statistical analysis or meta-analysis due to heterogeneity in the reported outcomes. Instead, all of the relevant study data—including incidence rates, odds ratios, and measures of central tendency (mean ± standard deviation, median [interquartile range]—were presented descriptively in the tables and summarized narratively in the main text.

## 3. Results

### 3.1. Study Selection and Characteristics

**Figure 1:**
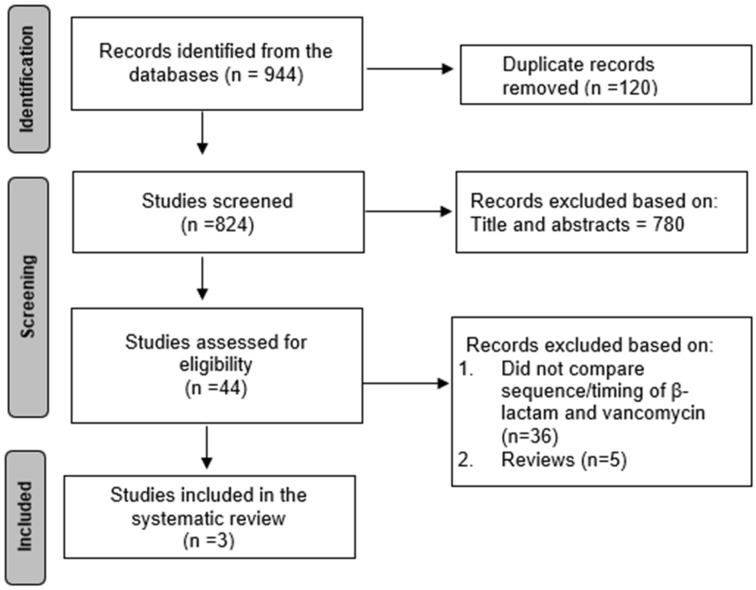
PRISMA flow diagram for selection of studies for systematic review.

The systematic search across the databases yielded a total of 944 studies (Figure 1). After removing the duplicates, 824 studies were screened based on titles and abstracts, and 780 studies were excluded for not meeting the eligibility criteria. A total of 44 studies were reviewed, and 41 were excluded due to a lack of reporting on the sequence or timing of beta-lactam and vancomycin administration (*n* = 36) or an inappropriate study type (*n* = 5). Therefore, a total of three retrospective observational studies evaluated the impact of the sequence or timing of beta-lactam and vancomycin administration on clinical outcomes in patients with bloodstream infections (BSIs). Patients with bloodstream infection were included in the systematic review. The total sample size of the patients in these studies was 29,005 patients, with 24,356 patients in the BLF and 4649 patients in the VF (Table 1). All studies were conducted in the United States and were multicenter studies except for one study, which was a single-center study. Regarding the mortality outcomes, one study evaluated 7-day and 48 h mortality outcomes from blood culture collection, and one study evaluated in-hospital mortality (Table 2). Other relevant information is shown in Table 1. The quality assessment scores for the studies ranged from 6 to 8, with two studies considered high-quality and one considered moderate quality (Table S3).

### 3.2. Summary of the Included Studies

The cumulative evidence from the included studies suggests that administering beta-lactam before vancomycin in patients with suspected or confirmed bloodstream infections (BSIs) may improve survival. Amoah and colleagues performed a multicenter retrospective cohort study of 3376 patients with confirmed BSIs [13]. A total of 3658 Gram-negative (66.3%) and 2476 Gram-positive (44.9%) organisms were reported. Common Gram-negatives included *E. coli* (20.8%), *K. pneumoniae* (13.9%), *P. aeruginosa* (6.9%), and *E. cloacae* (6.7%). Gram-positives were mainly methicillin-susceptible *S. aureus* (13.0%), MRSA (9.5%), *E. faecalis* (7.7%), and *E. faecium* (4.7%). The study reported that prioritizing beta-lactam over vancomycin resulted in a 52% reduction in the odds of 7-day mortality (adjusted OR, 0.48; 95% CI, 0.33–0.69) and a 55% reduction in the odds of 48 h mortality (adjusted OR: 0.45; 95% Cl, 0.24–0.83). However, no difference was observed between the groups in 7-day mortality among the subgroup of patients with MRSA-associated BSIs (adjusted OR: 0.93; 95% CI, 0.33–2.63). Similarly, Kondo et al. conducted a large multicenter retrospective cohort study of 25,391 patients with suspected sepsis and found that the BLF strategy was associated with a modest reduction in in-hospital mortality compared to the VF strategy (adjusted OR: 0.89; 95% CI: 0.80–0.99) [14]. However, no difference was found in the subgroups of patients with MRSA or septic shock (adjusted OR: 0.91; 95% CI: 0.81–1.03 and adjusted OR: 0.92; 95% CI: 0.81–1.05, respectively). When vancomycin was given first, the subsequent beta-lactam was administered a median of 164 min (~2.7 h) later, whereas when beta-lactam was given first, vancomycin followed after a median of 109 min (~1.8 h). Notably, patients in the VF were slightly younger, more often admitted to academic hospitals, and had more prior hospitalizations. They also had a lower comorbidity burden, fewer abnormalities in vital signs and lab values, and required less oxygen and vasopressor support within 12 h compared to the BLF. Cravero et al. conducted a single-institution study that included 238 patients with confirmed bacteremia [15]. The most frequently identified pathogens were *Escherichia coli* (25.2%) and *Staphylococcus aureus*, with MRSA at 13.5% and MSSA at 11.3%. Other notable organisms included *Proteus* spp. (7.6%), *Enterococcus faecalis* (5.9%), and *Klebsiella pneumoniae* (4.6%). Less common pathogens (<4% each) included various *Streptococcus* species, *Pseudomonas aeruginosa*, *Citrobacter*, *Pasteurella*, and anaerobes such as *Bacteroides* spp. Overall, Gram-negative bacteria predominated, but clinically relevant Gram-positive and resistant organisms were also present. The study reported a non-statistically significant trend towards lower 30-day mortality with the BLF approach (OR 0.40; 95% CI 0.089–1.831), attributing the lack of significance to the limited sample size.

## 4. Discussion

Observational studies examining the effect of empirical antibiotic sequencing on mortality in patients with suspected or confirmed bloodstream infections were combined into this systematic review. A decrease in mortality was associated with administering beta-lactam antibiotics prior to vancomycin in the largest multicenter studies [13,14]. However, in the most recent single-center study of patients with confirmed bacteremia, a statistically nonsignificant but clinically meaningful trend toward benefit was observed in BLF.

Fundamental pharmacodynamic and microbiological principles support the observed survival benefit. The main justification is that a BLF approach offers more rapid and efficient coverage for the most common and fatal infections. According to one included study, Gram-negative bacteria are responsible for 66.3% of infections, making them a very common cause of BSIs [13]. On the other hand, the main target of empiric vancomycin, methicillin-resistant *Staphylococcus aureus* (MRSA), accounts for a smaller percentage of cases, varying between 3.2% and 13.5% across cohorts [14,15]. Furthermore, the rapid inflammatory cascade caused by endotoxin release by Gram-negative BSIs is responsible for a high rate of early mortality [18,19]. Moreover, vancomycin needs a slower infusion of at least 60 min to avoid infusion reactions, while beta-lactam antibiotics can usually be infused efficiently in 15 to 30 min [20,21]. Accordingly, giving vancomycin first can cause a significant delay in effective treatment; one study measured this delay and discovered that the VF received beta-lactams after a median of 3.5 h later [14]. The benefit of treating the likely pathogen outweighs the risk of postponing therapy for the potential pathogen, as evidenced by the fact that two of the included studies specifically examined the MRSA-positive subgroup and found no evidence of harm or increased mortality from delaying vancomycin [13,14].

The findings of the study are consistent with the core principle of current sepsis management, which holds that one of the most important factors influencing survival is the time to effective antibiotic (TTE) [12,22]. Evidence has shown that patients experiencing septic shock had a 7.6% decrease in survival rate for every hour that effective antimicrobial therapy was delayed after hypotension started [5]. The 2021 Surviving Sepsis Campaign (SSC) guidelines mandate antibiotic administration “immediately, ideally within 1 h of recognition” for patients with probable sepsis or septic shock [12]. The studies in our review do not challenge this principle but rather refine it, addressing the more detailed clinical question of when simultaneous administration is not feasible. Kondo et al. found that a 3.5 h delay to beta-lactam in the VF was associated with an 11% increase in mortality odds [14]. While guidelines emphasize broad-spectrum combination therapy, they do not provide insights into the importance of the sequence of antibiotic administration. Our review provides strong, synthesized evidence that the sequence matters. Given that Gram-negative bacteremia is associated with a higher incidence of severe sepsis and a more intense inflammatory response, and that beta-lactams are the primary therapy for these pathogens, the findings from this review strongly support a sequential strategy that prioritizes the BLF strategy. This approach rapidly addresses the most probable and acutely lethal pathogens without incurring the 1 to 3.5 h therapeutic delay that a VF strategy requires.

The subgroup analyses within the included studies provide critical insights into the variability of the treatment effect. The most significant issue with a BLF strategy is the risk of delaying effective therapy for MRSA. Two of the included studies evaluated their MRSA-positive subgroups and found no significant difference in mortality [13,14]. These findings suggest that delaying vancomycin by one to two hours is not associated with increased mortality, even in MRSA subgroups. On the other hand, Cravero et al. reported that all five MRSA-associated deaths occurred in their BLF; however, this is an unadjusted finding that is confounded by patient age, comorbid conditions, and transitions to comfort care [15]. Variability was also observed in relation to illness severity. Kondo et al. reported that the mortality benefit of the BLF strategy was statistically significant only in patients with sepsis without shock [14]. This appears to contradict prior studies and guidelines, which identify septic shock as the condition where time-to-antibiotics is most critical [5,12]. However, the authors hypothesized that this was not due to a lack of benefit but rather to receiving all antibiotics much more rapidly, which minimized the difference in time to beta-lactam between the two sequences and thus masked the effect.

This is the first systematic review to synthesize the emerging evidence on this specific clinical question. However, it is necessary to acknowledge a few limitations. The retrospective and observational methodology of the included studies, which poses an inherent risk of residual confounding due to unmeasured variables, was a significant limitation. Second, a direct comparison or meta-analysis becomes more difficult due to the substantial heterogeneity among the studies. Furthermore, the primary mortality outcomes were heterogeneous, defined as 7-day, 30-day, and in-hospital mortality. The study populations differed substantially, ranging from a broad “suspected sepsis” cohort to specific “confirmed bacteremia” cohorts. Furthermore, the included studies did not assess vancomycin MICs, which can influence outcomes in bacteremic infections, potentially affecting the interpretation of our conclusions. Additionally, all studies reported all-cause mortality as the primary outcome at various time points, making it difficult to distinguish deaths directly due to sepsis from those caused by other conditions. Moreover, the findings of this review may not be generalizable to all clinical settings or regions, as practices regarding empirical antibiotic sequencing can vary. Most of the included studies had a high rate of Gram-negative bacteremia; therefore, the observed outcome differences between the groups may reflect delayed appropriate empiric beta-lactam therapy rather than a true sequencing effect. Finally, the statistical power varied widely; the Cravero et al. study was a single-center analysis with only 16 patients in the VF, rendering it underpowered to detect a statistically significant effect [15]. Despite these limitations, our review highlights the importance of the timely administration of broad-spectrum beta-lactams in patients with suspected or confirmed BSIs. Sequential addition of vancomycin without a clear indication or clinical suspicion of MRSA may delay optimal therapy and does not appear to improve outcomes. Vancomycin should therefore be reserved for cases with suspected or confirmed MRSA infections.

## 5. Conclusions

The evidence suggests a potential survival benefit for prioritizing beta-lactam administration over vancomycin in patients with suspected or confirmed BSIs. This finding is reasonable, as it provides faster coverage for common Gram-negative pathogens. Nevertheless, larger prospective studies are required to confirm the findings.

## Figures and Tables

**Table 1:** Summary of studies that were included in the systematic review.

First Author	Country	Year	Type	Number of Hospitals	BLF	VF	Population	Bacterial Organisms	Illness or Mortality Score		Age, in yrs: Mean (SD) Median (IQR)		Gender, in Number and %:	
									BLF	VF	BLF	VF	BLF	VF
Amoah [13]	USA	2021	Multicenter retrospective	5	2685 (79.5%)	691 (20.5%)	Patients ≥ 13 years with BSIs who received both agents within 6 h of blood culture	Gram-negative organisms 66.3% Gram-positive organisms 44.9% MRSA 9.5%	PBS 2 (1–3) Charlson comorbidity index 7 (3–10)	PBS 2 (1–3) Charlson comorbidity index 7 (3–10)	65 (53–76)	64 (54–76)	Female, *n* = 1159 (55.3%)	Female, *n* = 296 (54.9%)
Kondo [14]	USA	2024	Multicenter retrospective	5	21,449 (84.4%)	3942 (15.6%)	Adults with suspected sepsis who received both agents within 24 h and acute organ dysfunction within 12 h	More MRSA (4.5% vs. 3.2%; *p* < 0.001) in the VF	AHRQ Elixhauser mortality score 15 (1–30)	AHRQ Elixhauser mortality score 14 (1–28)	67 (56–78)	65 (53–73)	Female, *n* = 12,323 (57.5%)	Female, *n* = 2275 (57.7%)
Cravero [15]	USA	2025	Single-institution Retrospective	1	222 (93.3%)	16 (6.7%)	Patients > 18 years with confirmed bacteremia who received both agents within 6 h of admission	*Escherichia coli* 25.21% MRSA 13.45% MSSA 11.34%	NR	NR	66.7 ± 16.7 years		Female, *n* = 83 (37.39%)	Female, *n* = 6 (37.5%)

Abbreviations: AHRQ, Agency for Healthcare Research and Quality; BLF, β-lactam–first group; VF, vancomycin–first group; BSI, bloodstream infection; MRSA, methicillin-resistant ***Staphylococcus aureus***; MSSA, methicillin-susceptible ***Staphylococcus aureus***; PBS, Pitt bacteremia score; SD, standard deviation; IQR, interquartile range; NR, not reported.

**Table 2:** Comparison of mortality outcomes between BLF and VF.

First Author, Year, Country	Definition of Mortality	Incidence of Mortality		Crude Odds Ratio with 95% Confidence Interval (Cl)	Adjusted Odds Ratio (aOR) with 95% Confidence Interval (CI)
		BLF	VF		
Amoah 2021 USA [13]	Mortality within 7 days from blood culture collection	6.9% (186/2685)	10.7% (74/691)		
				OR: 0.68 [95% Cl, 0.50 –0.92]	aOR: 0.48 [95% Cl, 0.33–0.83]
					*p* = 0.001
					MRSA Subgroup
				NR	aOR: 0.93 [95% CI, 0.33–2.63]
	Mortality within 48 h from the time of blood culture collection	NR	NR	NR	aOR: 0.45 [95% Cl, 0.24–0.83]
Kondo 2024 USA [14]	In-hospital mortality	13.4% (2874/21,449)	13.6% (538/3942)		
				OR: 0.98 [95% Cl, 0.89 –1.08]	aOR: 0.89 [95% CI: 0.80–0.99]
	
					MRSA Subgroup
				NR	aOR: 0.91 [95% CI, 0.81–1.03]
Cravero 2025 USA [15]	30-day mortality	26.1% (58/222)	12.5% (2/16)	OR: 0.40 [95% CI: 0.089–1.831]	NR

Abbreviations: OR, odds ratio; aOR, adjusted odds ratio; BLF, β-lactam–first group; VF, vancomycin–first group; CI, confidence interval; MRSA, methicillin-resistant *Staphylococcus aureus*; NR, not reported.

## Data Availability

All data generated or analyzed during this study are included in this article and its Supplementary Materials.

## Supplementary Materials

The following supporting information can be downloaded at https://www.mdpi.com/article/10.3390/jcm15031024/s1. Table S1: PRISMA 2020 Checklist, Table S2: Keywords and search results, and Table S3: Newcastle–Ottawa scale for assessing the quality of included studies.
